# Independent and joint effects of high-sensitivity c-reactive protein and hypoalbuminemia on long-term all-cause mortality among coronary artery disease: a prospective and multicenter cohort study

**DOI:** 10.1186/s12872-021-02431-6

**Published:** 2021-12-27

**Authors:** Haozhang Huang, Yaren Yu, Liling Chen, Shiqun Chen, Ronghui Tang, Qiang Li, Wen Wei, Kunming Bao, Zhidong Huang, Wenguang Lai, Bo Wang, Ning Tan, Jiyan Chen, Jin Liu, Yong Liu

**Affiliations:** 1 Department of Cardiology, Guangdong Cardiovascular Institute, Guangdong Provincial People’s Hospital, Guangdong Academy of Medical Sciences, Guangzhou, 510080 China; 2 Department of Guangdong Provincial Key Laboratory of Coronary Heart Disease Prevention, Guangdong Cardiovascular Institute, Guangdong Provincial People’s Hospital, Guangdong Academy of Medical Sciences, Guangzhou, 510080 China; 3 The Second School of Clinical Medicine, Southern Medical University, Guangzhou, 510515 China; 4 The first people’s hospital of Foshan, No.81 of Lingnan Road, Chancheng District, Foshan, 528000 China; 5 Department of Cardiology, Longyan First Hospital Affiliated with Fujian Medical University, Longyan, 364000 China; 6 Yunnan Fuwai Cardiovascular Hospital, Department of Ultrasound Imaging, Yunnan, 650000 China; 7 Guangdong Provincial People’s Hospital Provincial People’s Hospital, School of Medicine, South China University of Technology, Guangzhou, 510100 China

**Keywords:** High-sensitivity C-reactive protein, Hypoalbuminemia, Long-term all-cause mortality, Coronary artery disease

## Abstract

**Background:**

High-sensitivity C-reactive protein (hs-CRP) plays an important role in hypoalbuminemia as a representative of inflammation, which is closely associated with poor prognosis among patients with coronary artery disease (CAD). The present study aimed to evaluate the independent and joint effects of high hs-CRP levels and hypoalbuminemia on long-term mortality among CAD patients.

**Methods:**

A total of 1449 CAD patients were included from a prospective, multicenter, observational cohort study (REICIN, NCT01402232) of patients referred for coronary angiography (CAG). The primary endpoint was long-term all-cause death.

**Results:**

During a median follow-up of 2.9 (2.0–3.0) years, a total of 107 (7.4%) patients died. The long-term mortality was higher among CAD patients with high hs-CRP levels (> 3 mg/L) than those with the low hs-CRP levels (≤ 3 mg/L; 10.7% versus 4.1%; hazard ratio [HR] 2.49; 95% confidence interval [CI] 1.48–4.17). Similarly, CAD patients with hypoalbuminemia had higher mortality than those without hypoalbuminemia (12.2% versus 4.9%; HR 1.93; 95% CI 1.20–3.08). When hs-CRP and albumin were combined, CAD patients with high hs-CRP levels (> 3 mg/L) and with hypoalbuminemia were at the highest risk of death compared with their reference group (hs-CRP ≤ 3 mg/L and albumin > 35 g/L; HR 3.79; 95% CI 1.91–7.52).

**Conclusions:**

High hs-CRP levels and hypoalbuminemia were independently and jointly associated with long-term mortality among CAD patients. Patients with high hs-CRP levels and hypoalbuminemia had the highest risk of long-term mortality compared with other groups.

**Supplementary Information:**

The online version contains supplementary material available at 10.1186/s12872-021-02431-6.

## Background

The Global Burden of Disease study indicated that coronary artery disease (CAD) remains the leading cause of morbidity and mortality [1]. Current studies emphasize the clinical importance of evaluating the prognosis of patients with CAD by using simple indicators.

Increasing evidence has shown that chronic inflammatory reaction, which is clinically presented as high-sensitivity C-Reactive Protein (hs-CRP), plays an important role in the development of CAD. Therefore, it is feasible for hs-CRP to predict adverse clinical events among CAD patients. Accordingly, both Momiyama et al. and Kim et al. have indicated that higher hs-CRP levels were found to be associated with a significantly increased risk for further cardiovascular events [2–4]. However, data concerning the association between hs-CRP and the risk of long-term mortality are limited.

Serum albumin, a major protein found in the extracellular fluid compartment, contributes to maintaining diverse physiological functions, such as anti-inflammatory, antioxidant, anticoagulant, and antiplatelet aggregation activity, as well as a colloid osmotic effect [5]. Further, studies have demonstrated that hypoalbuminemia is associated with poor survival in CAD patients [6, 7]. Interestingly, there is also a strong association between hypoalbuminemia and inflammation, which may be driven by the acute-phase response [8–10]. For example, inflammation is a driving force of reduced albumin levels, while hypoalbuminemia reflects the physiological stress from inflammation [11–13]. Notably, the Glasgow Prognostic Score, which calculated via serum albumin and inflammation indicators, and has been widely used in the field of oncology, has been shown to accurately predict the poor prognosis of patients with tumors in many studies [14–16].

However, the joint effect of hs-CRP and hypoalbuminemia among CAD patients remains unclear. Therefore, the current study aimed to test whether high hs-CRP levels can, independently and jointly with hypoalbuminemia, predict an increased risk of all-cause mortality among CAD patients in a multicenter prospective Reduction of Risk for Contrast-Induced Nephropathy (REICIN) study.

## Population, materials, and methods

### Data sources and study population

**Fig. 1:**
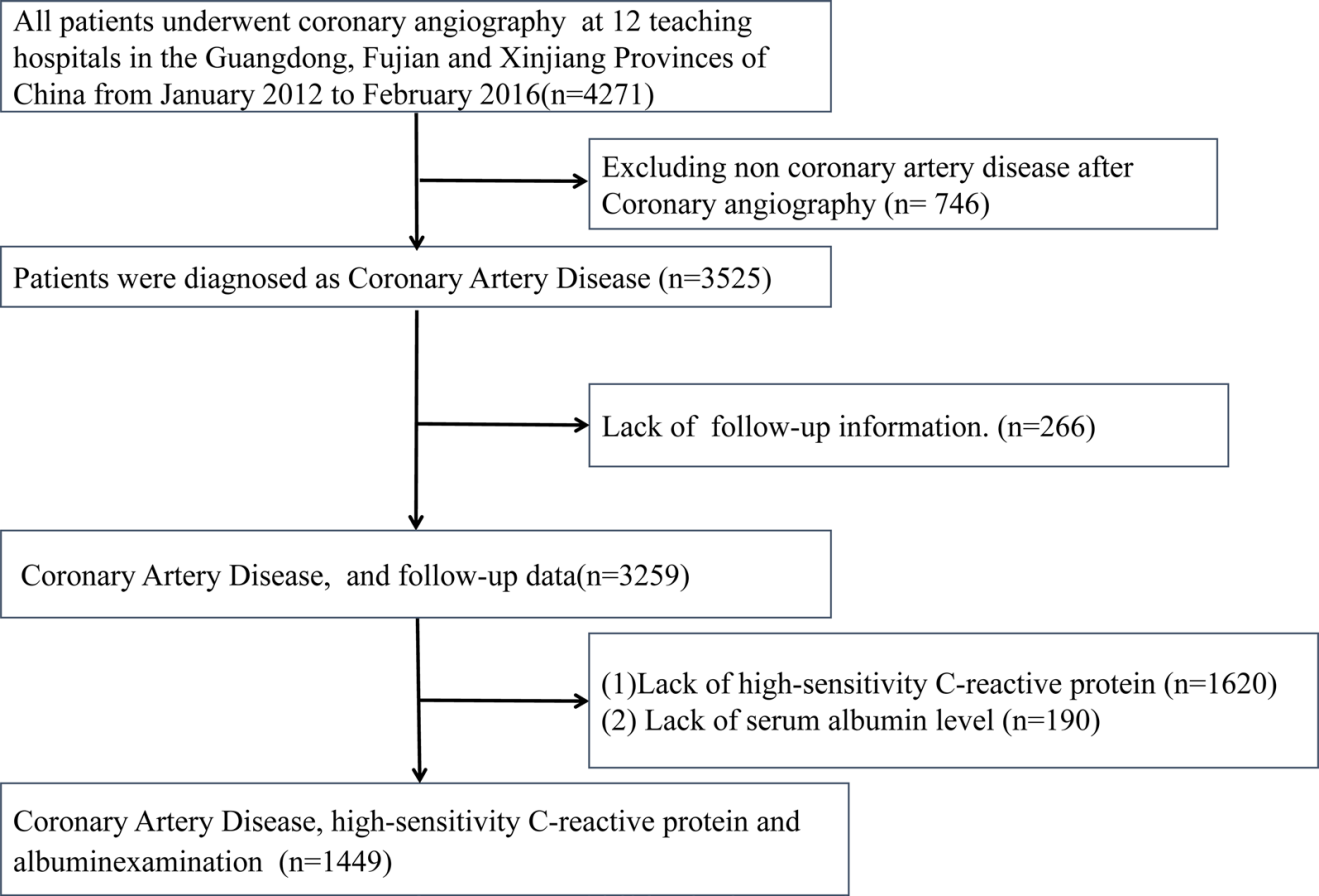
Flow of participants through the trial

Based on REICIN study data, the current study is a prospective, multicenter, observational cohort study to assess a method of reducing risk for contrast-induced nephropathy among patients given coronary angiography (CAG) who were admitted to one of 12 teaching hospitals in the Guangdong, Fujian, and Xinjiang Provinces of China from January 2013 to February 2016. Details of the site investigators and hospitals are presented in Additional file 1: Table S1. Patients were recruited according to the inclusion and exclusion criteria (Additional file 1: Table S2). Details of the cohort procedure are provided in Additional file 1: Fig. S1 and the inclusion/exclusion criteria are described in Additional file 1: Table S2. Based on the analysis of 4271 patients given CAG, the exclusion criteria were: patients who did not have a diagnosis of CAD (n = 746); patients who were missing follow-up information (n = 266); and patients whose data for serum levels of hs-CRP and albumin were missing (n = 1810). After exclusions, a total of 1449 CAD patients were enrolled (Fig. 1).

### Measurements of albumin and hs-CRP

Blood samples, including serum albumin, creatinine, glucose, hs-CRP, and other hematologic parameters, were obtained from all patients the morning after admission. Serum albumin levels were measured by the photometric method in the hospital laboratory. We used a high-sensitivity CRP enzyme-linked immunosorbent assay (ELISA) kit to determined hs-CRP levels. Both hs-CRP and serum albumin were detected in the same laboratory and the same measurement methods were used in all centers.

### Clinical definition

CAD was defined as a chronic coronary syndrome or acute coronary syndrome [17–19]. Chronic kidney disease (CKD) was defined as an estimated glomerular filtration rate (eGFR) < 60 mL/min/1.73 m^2^. eGFR was calculated by the Modification of Diet in Renal Disease (MDRD) equation [20]. Anemia was defined according to the World Health Organization criteria, i.e. baseline hematocrit value < 39% for men and < 36% for women. Hypoalbuminemia was defined as albumin < 35 g/L.

### Study endpoints and clinical follow-up

The primary endpoint was long-term all-cause mortality, which was defined as any death after the date of enrollment. This information was monitored and recorded by research assistants and trained nurses through outpatient interviews and via telephone. The proportion of subjects that were unable to complete follow-up did not exceed 10%.

### Statistical analysis

Descriptive statistics are reported as the mean (SD), median (interquartile range [IQR]) for continuous variables, and proportions for categorical variables. Differences between groups were analyzed with Student’s t-tests and one-way analyses of variance (ANOVAs) as appropriate. Categorical data were analyzed by Pearson chi-squared tests. We used log-transformation (log10) for describing hs-CRP concentration and used a restricted cubic splines (RCS) model to examine the associations between albumin/log10 hs-CRP and outcomes. The cut-offs for hs-CRP (≤ 3, or > 3 mg/L) and albumin (< 35, or ≥ 35 g/L) were based on previous studies [21–25]. We divided patients into four groups: Group 1 (non-hypoalbuminemia and hs-CRP ≤ 3 mg/L; Group 2 (hypoalbuminemia and hs-CRP ≤ 3 mg/L), Group 3 (non-hypoalbuminemia and hs-CRP > 3 mg/L), and Group 4 (hypoalbuminemia and hs-CRP > 3 mg/L). To estimate the effect of different groups on long-term all-cause mortality, Kaplan–Meier curves were constructed. Additionally, receiver-operator characteristic (ROC) curves were used to identify the optimal sensitivity for the observed range of albumin and hs-CRP among the four groups (Additional file 1: Fig. S2). Differences in survival rates were tested with the log-rank test. We also used density curves to assess the distribution of hs-CRP among acute coronary syndrome (ACS) and choric CAD (Additional file 1: Fig. S3).

Cox proportional hazard models were used to investigate the independent and joint effects of hs-CRP and albumin on long-term all-cause death among CAD patients. Hazard ratios and 95% CIs are reported. In the Cox hazard model, albumin and hs-CRP/log10 hs-CRP were presented as continuous and their tertiles (Table 2 and Additional file 1: Fig. S4). According to clinical experience and current studies, we adjusted the covariates in multivariate model, including demographic characteristics [age > 65, gender, and smoking], diagnosis [hypertension, chronic kidney diseases, acute myocardial infarction, stroke, diabetes mellitus, percutaneous coronary intervention, ejection fraction reduced heart failure, hyperlipidemia, and anemia], and medical information [angiotensin-converting enzyme inhibitor/angiotensin receptor blockers, β-blockers, and statins] [26–28]. Multivariate and interaction test was used to analyze the joint effect of hs-CRP and albumin on mortality (Table 3). We also performed a subgroup analysis among four prespecified subgroups (ACS or Choric CAD, older [age > 65] or younger [age ≤ 65], male or female, anemia or non-anemia) to assess the independent and joint effects of hs-CRP and albumin on long-term all-cause mortality and calculated the P interaction to assess the relationship between the endpoints and subgroups. Because of relatively small sample size, adjustment for multivariate not performed (Additional file 1: Table S4).

### Sensitivity analysis

We proceed a sensitivity analysis that those with severe hypoalbuminemia (≤ 25 g/L) (n = 7) are excluded from our included subjects to see whether the results were consistent. In addition, we also divided patients into four groups: group 1 (non-hypoalbuminemia and age ≤ 65; group 2 (hypoalbuminemia and age ≤ 65), group 3 (non-hypoalbuminemia and age > 65), and group 4 (hypoalbuminemia and age > 65). All data analyses were performed using R (version 4.0.3; R Core Team, Vienna, Austria). Two-tailed *P* values < 0.05 were considered statistically significant.

## Results

### Clinical characteristics

A total of 1449 patients were included in the study. Based on albumin levels (< 35 or ≥ 35 g/L), patients were divided into two groups; 501 (34.6%) had hypoalbuminemia and 948 (65.4%) did not have hypoalbuminemia. Under different albumin level groups, patients were further divided into two groups according to hs-CRP level (≤ 3 or > 3 mg/L). The mean overall age was 63.5 ± 10.8 years, and 329 (22.7%) were female. A total of 1041 (71.8%) patients underwent percutaneous coronary intervention (PCI) treatment, 317 (21.9%) patients were identified as having CKD, 426 (29.4%) patients had diabetes mellitus (DM), and 98 (7.6%) had ejection fraction reduced heart failure (EFrHF). In the hypoalbuminemia group, the mean age overall was 66.7 ± 9.5 years, and 127 (25.3%) were female. A total of 378 (75.4%) patients underwent PCI treatment, 159 (31.7%) patients were identified as having CKD, 157 (31.3%) patients had DM, and 61 (13.1%) had EFrHF. More data on the baseline characteristics of the study population are detailed in Table 1 and Additional file 1: Table S3.

**Table 1:** Baseline characteristics of the different albumin level

variable	Hypoalbuminemia^a^			*P* value	Non-hypoalbuminemia			*P* value
	Overall	Low hs-CRP	High hs-CRP^b^		Overall	Low hs-CRP	High hs-CRP	
n	501	138	363		948	580	368	
*Demographic characteristics*								
female	127 (25.3)	36 (26.1)	91 (25.1)	0.91	202 (21.3)	132 (22.8)	70 (19.0)	0.20
Age, years	66.7 (9.5)	67.57 (9.0)	66.4 (9.7)	0.23	61.8 (11.1)	62.3 (10.5)	61.2 (11.8)	0.14
Smoking	218 (43.5)	59 (42.8)	159 (43.8)	0.91	401 (42.3)	224 (38.6)	177 (48.1)	0.005
*Medical history*								
DM	157 (31.3)	37 (26.8)	120 (33.1)	0.22	269 (28.4)	157 (27.1)	112 (30.4)	0.30
Anemia	249 (50.0)	67 (48.9)	182 (50.4)	0.84	213 (22.6)	130 (22.6)	83 (22.7)	1
PCI	378 (75.4)	99 (71.7)	279 (76.9)	0.28	663 (69.9)	384 (66.2)	279 (75.8)	0.002
Hypertension	297 (59.3)	88 (63.8)	209 (57.6)	0.25	563 (59.4)	339 (58.4)	224 (60.9)	0.50
Hyperlipidemia	53 (10.6)	18 (13.0)	35 (9.6)	0.35	129 (13.6)	64 (11.0)	65 (17.7)	0.005
AMI	193 (38.5)	25 (18.1)	168 (46.3)	< 0.001	203 (21.4)	89 (15.3)	114 (31.0)	< 0.001
ACS	282 (56.3)	248 (42.8)	50 (36.2)	< 0.001	435 (45.9)	187 (50.8)	232 (63.9)	0.002
EFrEF	61 (13.6)	9 (7.9)	52 (15.6)	0.057	37 (4.4)	19 (3.6)	18 (5.5)	0.27
CKD	159 (31.7)	33 (23.9)	126 (34.7)	0.027	158 (16.7)	97 (16.7)	61 (16.6)	1
Stroke	30 (6.0)	6 (4.3)	24 (6.6)	0.46	38 (4.0)	20 (3.4)	18 (4.9)	0.35
*Laboratory tests*								
eGFR, mL/min/1.73 m^2^	72.8 (27.7)	77.7 (36.4)	71.0 (23.5)	0.016	82.3 (25.0)	81.9 (21.9)	82.8 (29.3)	0.58
LVEF, %	55.8 (13.2)	58.8 (12.1)	54.8 (13.4)	0.005	60.9 (10.1)	61.9 (9.6)	59.3 (10.7)	< 0.001
HDLC, mmol/L	0.92 (0.25)	0.97 (0.24)	0.90 (0.26)	0.007	0.99 (0.23)	1.00 (0.23)	0.97 (0.22)	0.02
LDLC, mmol/L	2.7 (1.0)	2.4 (0.9)	2.8 (1.0)	< 0.001	2.7 (1.1)	2.6 (1.0)	3.0 (1.2)	< 0.001
albumin, g/L	32.1 (2.5)	33.0 (1.5)	31.7 (2.7)	< 0.001	38.74 (2.97)	39.1 (3.3)	38.2 (2.3)	< 0.001
hs-CRP, mg/L	22.2 (35.8)	1.2 (0.8)	30.2 (39.2)	< 0.001	6.02 (14.10)	1.1 (0.8)	13.7 (20.4)	< 0.001
*Medications*								
ACEI/ARB	256 (51.1)	68 (49.3)	188 (51.8)	0.69	435 (45.9)	263 (45.3)	172 (46.7)	0.72
Beta-blockers	283 (56.5)	63 (45.7)	220 (60.6)	0.004	472 (49.8)	286 (49.3)	186 (50.5)	0.76
Clopidogrel	245 (48.9)	53 (38.4)	192 (52.9)	0.005	441 (46.5)	262 (45.2)	179 (48.6)	0.37
statin	219 (43.7)	46 (33.3)	173 (47.7)	0.005	396 (41.8)	228 (39.3)	168 (45.7)	0.063
aspirin	207 (41.3)	43 (31.2)	164 (45.2)	0.006	342 (36.1)	202 (34.8)	140 (38.0)	0.35

Values are means ± SDs, medians [IQRs], or n (%)

DM, diabetes mellitus; PCI, percutaneous coronary intervention; AMI, acute myocardial infarction; ACS, acute coronary syndrome; EFrEF, ejection fraction reduced heart failure; CKD, Chronic kidney disease; eGFR, estimated glomerular filtration rate; LDL-C,low-density lipoprotein cholesterol; HDL-C, Hight-density lipoprotein cholesterol; hs-CRP, High-Sensitivity C-Reactive Protein; ACEI/ARB, angiotensin-converting enzyme inhibitor/angiotensin receptor blocker

^a^Hypoalbuminemia stands for values of albumin < 35 g/L

^b^High hs-CRP stands for values of hs-CRP > 3 mg/L

### Primary outcomes

**Fig. 2:**
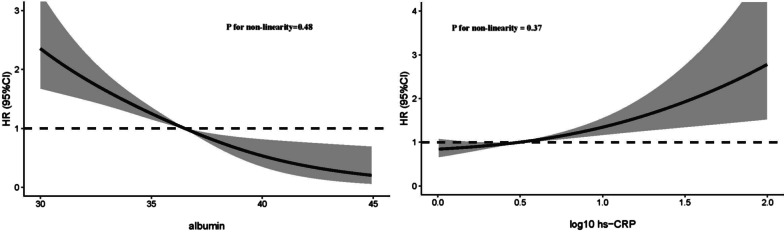
Restricted spline curve for the albumin and hs-CRP hazard ratio. **A** albumin; **B** log 10 hs-CRP

During a median follow-up of 2.9 years (interquartile range: 2.0 to 3.0 years), a total of 107 (7.4%) patients died. As can be seen in Fig. 2, there was a significant linear association between both hs-CRP and albumin with the risk of all-cause death. As you can see in Table 2, when albumin was a continuous variable, it was significantly associated with long-term mortality (HR 0.90, 95% CI 0.85–0.95, *P* < 0.001), whereas the result of log10 (hs-CRP) was opposite (HR 1.51, 95% CI 1.09–2.09, *P* = 0.014). Subsequently, in the multivariate model, with full adjustment, the lowest albumin level (T1) was correlated with a greater risk of mortality with albumin at the highest level (T3; HR 2.55, 95% CI 1.29–5.02, *P* = 0.007, *P* for trend = 0.005); highest hs-CRP level (T3) was associated with the greater risk of mortality with the lowest hs-CRP level (T1; HR 2.10, 95% CI 1.17–3.78, *P* = 0.013, P for trend = 0.013).

**Table 2:** Cox proportional hazard ratios of different risk factors for long-term all-cause mortality

Risk factor	n	Events, n (%)	All-cause mortality HR (95% Cl), *P* Value	
			Univariate	Multivariate^a^
Albumin, g/L				
*Continuous*				
IQR (33.9–39.2)	1449	107 (7.4%)	0.86 (0.83–0.91), < 0.001	0.90 (0.85–0.95), < 0.001
*Categories*				
Group 1 (≥ 35)	948	46 (4.9%)	Ref	Ref
Group 2 (< 35)	501	61 (12.2%)	2.49 (1.69–3.66), < 0.001	1.93 (1.20–3.08), 0.006
*Tertiles (min–max)*				
T3 (38.1–64.3)	472	15 (3.2%)	Ref	Ref
T2 (34.8–38.1)	488	35 (7.2%)	2.15 (1.17–3.95), 0.014	1.73 (0.86–3.49), 0.12
T1 (18.7—34.8)	474	57 (12.0%)	3.69 (2.08–6.52), < 0.001	2.55 (1.29–5.02), 0.007
*P* for trend			< 0.001	0.005
hs-CRP, mg/L				
*Continuous*				
IQR (0.96–9.1)	1449	107 (7.4%)	1.01 (1.00–1.01), < 0.001	1.00 (1.00–1.01), 0.18
log10 (hs-CRP)	1449	107 (7.4%)	1.72 (1.31–2.26), < 0.001	1.51 (1.09–2.09), 0.014
*Categories*				
Group 1 (≤ 3)	716	29 (4.1%)	Ref	Ref
Group 1 (> 3)	731	78 (10.7%)	2.60 (1.69–3.99), < 0.001	2.49 (1.48–4.17), < 0.001
*Tertiles (min–max)*				
T1 (0.18–1.4)	481	23 (4.8%)	Ref	Ref
T2 (1.4–6.0)	481	27 (5.6%)	1.23 (0.71–2.15), 0.46	1.52 (0.81–2.88), 0.20
T3 (6.0–194)	485	57 (11.8%)	2.41 (1.48–3.93), < 0.001	2.10 (1.17–3.78), 0.013
P for trend			< 0.001	0.013

^a^Adjusted for full multivariate: age > 65, gender, smoking, hypertension, acute myocardial infarction, stroke, diabetes mellitus, percutaneous coronary intervention, ejection fraction reduced heart failure, hyperlipidemia, anemia, angiotensin-converting enzyme inhibitor/angiotensin receptor blockers, β-blockers, statins

**Fig. 3:**
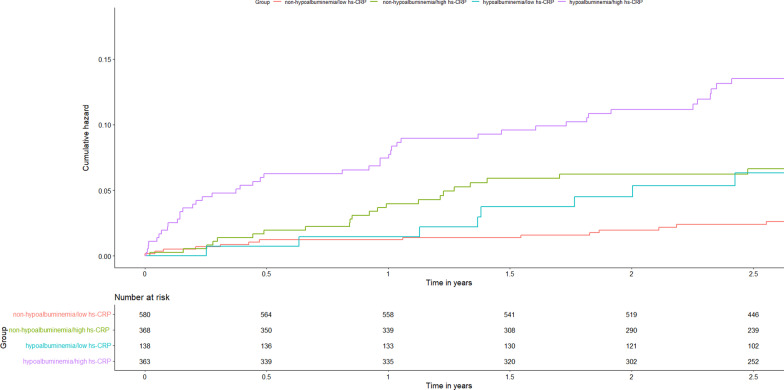
Kaplan–Meier curves of cumulative hazards of different hs-CRP levels (≤ 3 mg/L vs > 3 mg/L) and hypoalbuminemia (yes, no) on all-cause mortality

As can be seen in Fig. 3 and Table 3, when hs-CRP levels (≤ 3 mg/L vs. > 3 mg/L) and hypoalbuminemia (yes or no) were taken together, the Kaplan–Meyer curves showed that the cumulative hazard of death risk significantly differed among the four groups and that Group 4 (hs-CRP > 3 mg/L and hypoalbuminemia) had the highest risk of death when compared to the other groups (Group1 vs Group 2 vs Group 3 vs Group 4: 2.9% vs 7.9% vs 8.7% vs 13.5%). Cox proportional hazard regression analyses estimated that the highest risk of death was in Group 4 (hypoalbuminemia and hs-CRP > 3 mg/L) in multivariate model, (HR 3.79, 95% CI 1.91–7.52, *P* < 0.001). The analyses also revealed an additive effect of hypoalbuminemia and increased hs-CRP in predicting the risk of all-cause mortality. Further, results were consistent across all subgroups, even among the ACS (yes vs. no) and age groups (older [age > 65] vs. younger [age ≤ 65]; *P*-interaction > 0.05; Additional file 1: Table S4).

**Table 3:** Crude mortality and Cox proportional hazard ratios in different groups

Combined groups		Events, n (%)	All-cause mortality HR (95% Cl), *P* value		
Albumin level	hs-CRP level		Univariate	Multivariate^a^	*P* for interaction^a^
Non-hypoalbuminemia	Low hs-CRP	17 (2.9%)	Reference	Reference	0.04
	High hs-CRP^2^	29 (7.9%)	2.73 (1.50–4.97), 0.001	3.03 (1.48–6.19), 0.002	
Hypoalbuminemia	Low hs-CRP	12 (8.7%)	3.12 (1.49–6.56), 0.003	2.68 (1.11–6.46), 0.029	
	High hs-CRP	39 (13.5%)	4.51 (2.59–7.86), < 0.001	3.79 (1.91–7.52), < 0.001	

^a^Adjusted for full multivariate: age > 65, gender, smoking, hypertension, acute myocardial infarction, stroke, diabetes mellitus, percutaneous coronary intervention, ejection fraction reduced heart failure, hyperlipidemia, anemia, angiotensin-converting enzyme inhibitor/angiotensin receptor blockers, β-blockers, statins

### Sensitivity analysis

Our overall findings were consistent after performing the sensitivity analyses described in the methods (Additional file 1: Table S5). We note that the patients with hypoalbuminemia had lower HDLC level and more complications, such as anemia, AMI, CKD and more. However, in the group of age ≤ 65, there was no difference in some complications, and the incidence of hypoalbuminemia was significantly lower than that in the group of age > 65 (Additional file 1: Table S6).

## Discussion

We systematically assessed the significance of concomitant high hs-CRP levels and hypoalbuminemia on long-term all-cause mortality among CAD patients. We found that concomitant high hs-CRP levels (> 3 mg/L) and hypoalbuminemia (< 35 g/L) increased the risk of long-term all-cause mortality among CAD patients near four-fold.

Previous studies have shown that hs-CRP level, which is a biomarker of systemic inflammation, has been reported to predict cardiovascular events among a wide variety of population such as CAD patients [29–31]. For example, Momiyama et al. indicated that an hs-CRP level > 1.0 mg/L was an independent predictor for cardiovascular events in Japanese patients with stable CAD [3]. Kim et al. showed that elevated levels of hs-CRP (≥ 3 mg/L) was an independent predictor of long-term cardiovascular outcomes only among ST-elevated myocardial infarction (STEMI) patients with a long ischemic time[2]. While both of these studies are excellent proof-of-concepts, the absence of long-term all-cause death is a limitation. It should also be mentioned that studies have suggested inflammation may play a different role in ACS and stable CAD on various elements, such as cytokine levels and proinflammatory status [32]. Although the distribution of hs-CRP was different among ACS and stable CAD patients, the increased risk relation of combined hs-CRP to albumin was consistent, which may rely on the mechanism of inflammation in hypoalbuminemia.

Conversely, epidemiologic studies have reported that hypoalbuminemia concentration predicted adverse outcomes in several populations, such as individuals with ACS [33–35]. Touma et al. indicated patients with hypoalbuminemia were older, and with increased comorbidity, than patients with normal albumin levels [36]. The pathological process in these conditions can be inflammatory, degenerative, or malignant, and has been confirmed in various medical conditions, as shown in recent studies [37, 38].

The prognostic relevance of serum albumin in cardiovascular disorders primarily refers to hypoalbuminemia and inflammation. Wiedermann indicated that preexisting hypoalbuminemia contributes to the risk of acute infectious diseases, and that acute loss of albumin in systemic inflammatory reactions further complicates the clinical course of all trauma, medical, and surgical conditions [12]. The high-sensitivity Modified Glasgow Prognostic Score are measures that utilize hs-CRP and albumin levels, and have been widely used in the field of oncology. Previous studies [39, 40] have demonstrated their ability in predicting poor prognosis in patients with tumors. CAD has a similar pathophysiological process to the tumor, which is related to inflammation and hypoalbuminemia. Our study indicated that a high hs-CRP level increases the risk of long-term all-cause mortality, especially in CAD patients with hypoalbuminemia. What is exciting is that our conclusion coincides with Hideki Wada, et al.’s study [27]. We included an interaction test in additive scale for the joint effect of hs-CRP and hypoalbuminemia on mortality. We found that the P for interaction between hs-CRP and albumin was 0.04. Our study indicated that, especially for CAD patients with high hs-CRP levels, hypoalbuminemia increased the risk of long-term all-cause mortality. Serum albumin concentration is defined by a complex interaction of several factors, including inflammation, nutrition, and dialysis efficacy. Therefore, the prognostic value of serum albumin is mainly explained by inflammation [11–13].

Furthermore, we noted that the population with hypoalbuminemia was likely to be older and had more complications. Our study showed among middle-aged subjects, risks of outcomes in individuals who had high CRP levels alone or in individuals who had low serum albumin levels alone attenuate or even lose statistical power. It indicated that exploring hypoalbuminemia and high CRP levels in the elderly has greater significance and more attention should be paid to the treatment of hypoalbuminemia and high CRP levels in the elderly in subsequent studies.

Our study showed that it was very meaningful to assess the degree of serum albumin and inflammation in patients with CAD. Better prognosis hinges on timely recognition of, and action toward, clinical clues. CAD patients with hypoalbuminemia must receive medical tests including computed tomography, urine analysis and more to determine the reason for albumin decrease, and subsequently adopt effective therapy. For example, when nutritional intake is lacking, a multidisciplinary nutrition team may give them nutritional support nutritional supplementation. Interdisciplinary approaches should be considered in these patients. Furthermore, some treatments that reduce CAD-related risks factors (such as smoking, hyperlipemia, hypertension, DM,, and overweight) can also reduce inflammation [41]. In addition, lipid-lowering therapy can reduce inflammation in atherosclerosis by severely controlling low-density lipoprotein cholesterol [42].

## Limitations

This study systematically examined the significance of concomitant hypoalbuminemia and high hs-CRP levels on long-term all-cause mortality among CAD patients. The abundant data extracted from multiple centers allowed us to control a variety of confounders during analyses. Nevertheless, there are several limitations to the current study. First, information about cause-specific mortality was not available in this study, which restricted our ability to examine the significance of concomitantly high hs-CRP levels and hypoalbuminemia with cause-specific mortality. Second, although hs-CRP and CRP are usually tested together in the clinical practice, we strictly did not include the patients without the data of hs-CRP, even if these patients had CRP. As a result, selection bias should be existed. However, there was no special tendency for clinicians to choose these two tests in general and the difference of mortality rates between excluded subjects and including subjects were no statistic significance. However, our study was based on a multicenter prospective cohort study including all CAD patients. Thus, future testing should be conducted on a larger population of CAD patients. Third, only inpatient information about baseline hs-CRP and albumin were contained in our study. Therefore, follow-up information of hs-CRP and albumin should be collected to better understand their status and the effects of changes after discharge. Fourth, we did not have access to several variables that may have influenced the final results, including body mass index and diet structure. Finally, the current study lacked corresponding information on malignant diseases that may cause protein-energy malnutrition and inflammation. Further studies that assess the role of malignant diseases are warranted to corroborate these results.

## Conclusions

High hs-CRP levels, as well as hypoalbuminemia, are effective prognostic factors of long-term all-cause mortality in CAD patients, especially in elderly patients. Importantly, CAD patients with high hs-CRP levels (> 3 mg/L) and hypoalbuminemia (< 35 g/L) exhibited a three-fold increased risk of long-term all-cause mortality. Clinically, prospective large scale population studies are necessary to more deeply explore high hs-CRP levels and hypoalbuminemia in CAD patients with a high risk of mortality.

## Supplementary Information

**Additional file 1: Supplementary instructions 1–10:** 1. Investigators or sub-investigators; 2. The inclusion criteria and exclusion criteria of REICIN cohort; 3. Baseline characteristics of the patients; 4. Crude mortality and Cox proportional hazard ratios of different subgroups; 5. Sensitivity analysis; 6. Characteristics between subjects with and without hypoalbuminemia; 7. The flow of participants through REICIN cohort; 8. receiver-operator characteristic (ROC) curves; 9. Density curve of hs-CRP among ACS and chronic CAD; 10. Dose-response relationship between hs-CRP tertiles and unadjusted hazard ratios and 95% confidence intervals of long-term all-cause mortality, stratified by status of albumin.

## Data Availability

The datasets used and analysed during the current study available from the corresponding author on reasonable request.

## Abbreviations

AMI: Acute myocardial infarction
CAD: Coronary artery disease
CKD: Chronic kidney disease
hs-CRP: High-sensitivity C-reactive protein
PCI: Percutaneous coronary intervention
