# Supplementary material for: Independent and joint effects of high-sensitivity c-reactive protein and hypoalbuminemia on long-term all-cause mortality among coronary artery disease: a prospective and multicenter cohort study
Source: BMC Cardiovasc Disord. 2021 Dec 27;21:613. doi: 10.1186/s12872-021-02431-6 (PMC8714430; doi:10.1186/s12872-021-02431-6)
Supplement: Supplementary file 1 — Additional file 1: Supplementary instructions 1–10: 1. Investigators or sub-investigators; 2. The inclusion criteria and exclusion criteria of REICIN cohort; 3. Baseline characteristics of the patients; 4. Crude mortality and Cox proportional hazard ratios of different subgroups; 5. Sensitivity analysis; 6. Characteristics between subjects with and without hypoalbuminemia; 7. The flow of participants through REICIN cohort; 8. receiver-operator characteristic (ROC) curves; 9. Density curve of hs-CRP among ACS and chronic CAD; 10. Dose-response relationship between hs-CRP tertiles and unadjusted hazard ratios and 95% confidence intervals of long-term all-cause mortality, stratified by status of albumin. [file 12872_2021_2431_MOESM1_ESM.docx]

**Supplementary Table 1 Investigators or sub-investigators.**

| No. | Investigators or sub-investigators | Institution |
| --- | --- | --- |
| 01 | Jiyan Chen, Ning Tan, Yingling Zhou, Yong Liu, Shiqun Chen, Jianfang Luo, Danqing Yu, Liwen Li, Zhujun Chen, Guang Li, Bin Zhang, Lijun Jin, Hong Yan, Taiming Dong, Yuan Liu, Wenhui Huang, Junqing Yang, Pengcheng He, Nianjin Xie. | Guangdong Provincial Key Laboratory of Coronary Disease, Guangdong Cardiovascular Institute, Guangdong Provincial People’s Hospital, Affiliated Guangdong Provincial People’s Hospital of South China University of Technology |
| 02 | Zhimin Du, Yi Li, Ming Long | The First Affiliated Hospital, Sun Yat-sen University |
| 03 | Jingfeng Wang, Ruqiong Nie | Sun Yat-sen Memorial Hospital, Sun Yat-sen University |
| 04 | Yuqing Hou, Jiancheng Xiu, Zheng Huang | Nanfang Hospital, South medical university |
| 05 | Jian Qiu, Dingcheng Xiang, Changjiang Hong | General Hospital of Guangzhou Military Command of People’s Liberation Army |
| 06 | Keng Wu, Chang Peng | Affiliated Hospital of Guangdong Medical University |
| 07 | Guoliang Jia, Qibing Cai | Dongguan Kanghua Hospital |
| 08 | Jianfeng Ye, Shaohui Su | Dongguan People's Hospital |
| 09 | Yan Liang, Cong Chen | Maoming People's Hospital |
| 10 | Guifu Wu, Jiansheng Wu | The Eighth Affiliated Hospital, Sun Yat-sen University |
| 11 | Kaihong Chen, Liling Chen | Longyan First Affiliated Hospital of Fujian Medical University |
| 12 | Xiaoguang Zhou, Yuying Hu, Haiyan Wei | First People's Hospital of Kashgar |

**Supplementary Table 2 The inclusion criteria and exclusion criteria of REICIN cohort.**

| Inclusion criteria | Exclusion criteria |
| --- | --- |
| (1) Subjects who were referred for CAG or PCI;  (2) Aged 18 years or over;  (3) Submitted informed consent, and complied with the study protocol; | (1) Fail to undergo CAG/PCI or death during the procedure;  (2) End-stage renal disease or renal replacement therapy;  (3) Unstable renal function prior to the procedure (acute increase in serum creatinine of more than 0.5 mg/ml in the previous 24 h);  (4) Intravascular administration of contrast medium within the previous 48 h;  (5) Allergy to the contrast medium;  (6) Pregnancy, lactation, or malignant tumor with a life expectancy of <1 year;  (7) Use of nephrotoxic drugs (non-steroidal anti-inflammatory drugs, aminoglycosides, cyclosporine, cisplatin, etc.) except for aspirin in the 48 hours prior to cardiac catheter surgery and throughout the research period;  (8) Renal artery angiography or surgical valve replacement in patients with rheumatic heart disease. |

**Supplementary Table 3 Baseline characteristics of the patients.**

| variable | Overall | Hypoalbuminemia ^2^ | | Non-hypoalbuminemia | | P value |
| --- | --- | --- | --- | --- | --- | --- |
|  |  | Low hs-CRP | High hs-CRP^3^ | Low hs-CRP | High hs-CRP |  |
|  | N=1449 | N=138 | N=363 | N=580 | N=368 |  |
| **Demographic characteristics** | | | | | | |
| female | 329 (22.7) | 36 (26.1) | 91 (25.1) | 132 (22.8) | 70 (19.0) | 0.179 |
| Age, years | 63.5 (10.8) | 67.57 (9.0) | 66.4 (9.7) | 62.3 (10.5) | 61.2 (11.8) | <0.001 |
| Smoking | 619 (42.7) | 59 (42.8) | 159 (43.8) | 224 (38.6) | 177 (48.1) | 0.037 |
| **Medical history** | | | | | | |
| DM | 426 (29.4) | 37 (26.8) | 120 (33.1) | 157 (27.1) | 112 (30.4) | 0.213 |
| Anemia | 462 (32.1) | 67 (48.9) | 182 (50.4) | 130 (22.6) | 83 (22.7) | <0.001 |
| PCI | 1041 (71.8) | 99 (71.7) | 279 (76.9) | 384 (66.2) | 279 (75.8) | 0.002 |
| Hypertension | 860 (59.4) | 88 (63.8) | 209 (57.6) | 339 (58.4) | 224 (60.9) | 0.5 |
| Hyperlipidemia | 1267 (87.4) | 18 (13.0) | 35 (9.6) | 64 (11.0) | 65 (17.7) | 0.005 |
| AMI | 396 (27.3) | 25 (18.1) | 168 (46.3) | 89 (15.3) | 114 (31.0) | <0.001 |
| ACS | 717 (49.5) | 248 (42.8) | 50 (36.2) | 187 (50.8) | 232 (63.9) | <0.001 |
| EFrHF | 98 (7.6) | 9 (7.9) | 52 (15.6) | 19 (3.6) | 18 (5.5) | <0.001 |
| CKD | 317 (21.9) | 33 (23.9) | 126 (34.7) | 97 (16.7) | 61 (16.6) | <0.001 |
| Stroke | 68 (4.7) | 6 (4.3) | 24 (6.6) | 20 (3.4) | 18 (4.9) | 0.167 |
| **Laboratory tests** | | | | | | |
| eGFR, mL/min/1.73 m^2^ | 79.0 (26.4) | 77.7 (36.4) | 71.0 (23.5) | 81.9 (21.9) | 82.8 (29.3) | <0.001 |
| HDLC, mmol/L | 0.96 (0.24) | 0.97 (0.24) | 0.90 (0.26) | 1.00 (0.23) | 0.97 (0.22) | <0.001 |
| LDLC, mmol/L | 2.7 (1.1) | 2.4 (0.9) | 2.8 (1.0) | 2.6 (1.0) | 3.0 (1.2) | <0.001 |
| albumin, g/L | 36.4 (4.2) | 33.0 (1.5) | 31.7 (2.7) | 39.1 (3.3) | 38.2 (2.3) | <0.001 |
| hs-CRP, mg/L | 11.6(25.1) | 1.2 (0.8) | 30.2 (39.2) | 1.1 (0.8) | 13.7 (20.4) | <0.001 |
| **Medications** | | | | | | |
| ACEI/ARB | 691 (47.7) | 68 (49.3) | 188 (51.8) | 263 (45.3) | 172 (46.7) | 0.262 |
| Beta-blockers | 755 (52.1) | 63 (45.7) | 220 (60.6) | 286 (49.3) | 186 (50.5) | 0.002 |
| Clopidogrel | 686 (47.3) | 53 (38.4) | 192 (52.9) | 262 (45.2) | 179 (48.6) | 0.017 |
| statin | 615 (42.4) | 46 (33.3) | 173 (47.7) | 228 (39.3) | 168 (45.7) | 0.006 |
| aspirin | 549 (37.9) | 43 (31.2) | 164 (45.2) | 202 (34.8) | 140 (38.0) | 0.004 |
| **Long-term all-cause mortality** | 107 (7.4) | 17 (2.9) | 12 (8.7) | 29 (7.9) | 49 (13.5) | <0.001 |

^1^Values are means ± SDs, medians [IQRs], or n (%);DM= diabetes mellitus; PCI = percutaneous coronary intervention; AMI = acute myocardial infarction; ACS = acute coronary syndrome; EFrEF = ejection fraction reduced heart failure; CKD= Chronic kidney disease; eGFR = estimated glomerular filtration rate; LDL-C =low-density lipoprotein cholesterol; HDL-C= Hight-density lipoprotein cholesterol; hs-CRP = High-Sensitivity C-Reactive Protein; ACEI/ARB = angiotensin-converting enzyme ^2^ Hypoalbuminemia stands for values of albumin < 35 g/L.

^3^High hs-CRP stands for values of hs-CRP > 3 mg/L.

**Supplement table 4** Crude mortality and Cox proportional hazard ratios of different subgroups.

| Long-term all-cause mortality | | | | | |  |  |
| --- | --- | --- | --- | --- | --- | --- | --- |
| Subgroup | albumin level | hs-CRP level | n | Events, n (%) | HR,95%Cl | P-value | P-interaction |
| Gender |  |  |  |  |  |  | 0.22 |
| Male | Non-hypoalbuminemia | Low hs-CRP | 448 | 12(2.7%) | reference | reference |  |
|  |  | High hs-CRP^2^ | 298 | 22(7.4%) | 2.7(1.3-5.5) | 0.007 |  |
|  | Hypoalbuminemia | Low hs-CRP | 102 | 8(7.8%) | 3.0(1.2-7.3) | 0.02 |  |
|  |  | High hs-CRP | 272 | 38(14.0%) | 5.3(2.7-10.1) | <0.001 |  |
| Female | Non-hypoalbuminemia | Low hs-CRP | 132 | 5(3.9%) | reference | reference |  |
|  |  | High hs-CRP^2^ | 70 | 7(10.0%) | 2.8(0.9-8.9) | 0.08 |  |
|  | Hypoalbuminemia | Low hs-CRP | 36 | 4(11.1%) | 3.4(0.9-12.7) | 0.07 |  |
|  |  | High hs-CRP | 91 | 11(12.1%) | 2.9(1.0-8.5) | 0.05 |  |
| older |  |  |  |  |  |  | 0.83 |
| Yes | Non-hypoalbuminemia | Low hs-CRP | 232 | 8(3.4%) | reference | reference |  |
|  |  | High hs-CRP^2^ | 134 | 20(14.9%) | 4.3(1.9-9.9) | <0.001 |  |
|  | Hypoalbuminemia | Low hs-CRP | 90 | 10(11.1%) | 3.3(1.3-8.4) | 0.01 |  |
|  |  | High hs-CRP | 200 | 34(17.0%) | 5.0(2.3-10.9) | <0.001 |  |
| No | Non-hypoalbuminemia | Low hs-CRP | 348 | 9(2.6%) | reference | reference |  |
|  |  | High hs-CRP^2^ | 234 | 9(3.8%) | 3.3(1.0-11.3) | 0.04 |  |
|  | Hypoalbuminemia | Low hs-CRP | 48 | 2(4.2%) | 2.9(0.6-16.7) | 0.22 |  |
|  |  | High hs-CRP | 163 | 15(9.2%) | 4.2(1.3-13.8) | 0.02 |  |
| ACS |  |  |  |  |  |  | 0.65 |
| Yes | Non-hypoalbuminemia | Low hs-CRP | 248 | 8(3.2%) | reference | reference |  |
|  |  | High hs-CRP^2^ | 187 | 11(5.9%) | 2.2(0.8-5.6) | 0.25 |  |
|  | Hypoalbuminemia | Low hs-CRP | 50 | 3(6.0%) | 2.2(0.6-8.6) | 0.11 |  |
|  |  | High hs-CRP | 232 | 26(11.2%) | 4.3(1.8-9.9) | <0.001 |  |
| No | Non-hypoalbuminemia | Low hs-CRP | 332 | 9(2.7%) | reference | reference |  |
|  |  | High hs-CRP^2^ | 181 | 18(9.9%) | 3.6(1.6-8.0) | 0.002 |  |
|  | Hypoalbuminemia | Low hs-CRP | 88 | 9(10.2%) | 3.9(1.6-9.9) | 0.004 |  |
|  |  | High hs-CRP | 131 | 23(17.6%) | 5.8(2.6-12.7) | <0.001 |  |
| Anemia |  |  |  |  |  |  | 0.21 |
| Yes | Non-hypoalbuminemia | Low hs-CRP | 130 | 5(3.8%) | reference | reference |  |
|  |  | High hs-CRP^2^ | 83 | 12(14.5%) | 3.4(1.2-9.8) | 0.02 |  |
|  | Hypoalbuminemia | Low hs-CRP | 67 | 8(11.9%) | 3.5(1.1-10.7) | 0.03 |  |
|  |  | High hs-CRP | 182 | 30(16.5%) | 4.9(1.9-12.6) | 0.001 |  |
| No | Non-hypoalbuminemia | Low hs-CRP | 446 | 12(2.7%) | Reference | reference |  |
|  |  | High hs-CRP^2^ | 282 | 17(6.0%) | 2.3(1.1-4.8) | 0.02 |  |
|  | Hypoalbuminemia | Low hs-CRP | 70 | 4(5.7%) | 2.2(0.7-6.8) | 0.17 |  |
|  |  | High hs-CRP | 179 | 19(10.1%) | 3.6(1.7-7.5) | <0.001 |  |

**Supplement Table 5** Sensitivity analysis

| **Combined Groups** | | **all-cause mortality HR (95%Cl), P Value** | | |  |
| --- | --- | --- | --- | --- | --- |
| albumin level | hs-CRP level | **model1**^*^ | **model2**^$^ | **model3**^§^ | **P for interaction**^§^ |
| Non-hypoalbuminemia | Low hs-CRP | reference | reference | reference | 0.04 |
|  | High hs-CRP^2^ | 2.73(1.50-4.97), 0.001 | 2.76(1.51-5.05), <0.001 | 3.03(1.48-6.19), 0.002 |  |
| Hypoalbuminemia | Low hs-CRP | 3.12(1.49-6.56), 0.003 | 2.48(1.18-5.21), 0.017 | 2.68(1.11-6.46), 0.029 |  |
|  | High hs-CRP | 4.51(2.59-7.86), <0.001 | 3.84(2.20-6.71), <0.001 | 3.79(1.91-7.52), <0.001 |  |

^*^Unadjusted

^$^Adjusted for age > 65 and gender

^§^Adjusted for full multivariate: age > 65, gender, smoking, hypertension, acute myocardial infarction, stroke, diabetes mellitus, percutaneous coronary intervention, ejection fraction reduced heart failure, hyperlipidemia, anemia, angiotensin-converting enzyme inhibitor/angiotensin receptor blockers, β-blockers, statins.

**Supplement Table 6** Characteristics between subjects with and without hypoalbuminemia

| variable | age ≤65 | | | age > 65 | | | P value | age ≤ 65 |
| --- | --- | --- | --- | --- | --- | --- | --- | --- |
|  | Non-hypoalbuminemia | hypoalbuminemia | P value | Non-hypoalbuminemia | hypoalbuminemia | P value |  |  |
|  | N=543 | N=193 |  | N=405 | N=308 |  |  |  |
| **Demographic characteristics** | | | | | | | |  |
| female | 463 (85.3) | 163 (84.5) | 0.878 | 283 (69.9) | 211 (68.5) | 0.756 | <0.001 |  |
| Age, years | 54.3 (7.6) | 57.1 (6.0) | <0.001 | 72.0 (5.4) | 72.8 (5.4) | 0.056 | <0.001 |  |
| Smoking | 274 (50.5) | 99 (51.3) | 0.908 | 127 (31.4) | 119 (38.6) | 0.052 | <0.001 |  |
| **Medical history** | | | | | | | |  |
| DM | 137 (25.2) | 62 (32.1) | 0.079 | 132 (32.6) | 95 (30.8) | 0.678 | 0.057 |  |
| Anemia | 82 (15.2) | 82 (42.7) | <0.001 | 131 (32.5) | 167 (54.6) | <0.001 | <0.001 |  |
| PCI | 396 (72.9) | 148 (76.7) | 0.355 | 267 (65.9) | 230 (74.7) | 0.015 | 0.013 |  |
| Hypertension | 271 (49.9) | 103 (53.4) | 0.458 | 292 (72.1) | 194 (63.0) | 0.012 | <0.001 |  |
| Hyperlipidemia | 87 (16.0) | 24 (12.4) | 0.281 | 42 (10.4) | 29 (9.4) | 0.768 | 0.015 |  |
| AMI | 130 (23.9) | 96 (49.7) | <0.001 | 73 (18.0) | 97 (31.5) | <0.001 | <0.001 |  |
| ACS | 265 (48.8) | 122 (63.2) | 0.001 | 170 (42.0) | 160 (51.9) | 0.01 | <0.001 |  |
| EFrHF | 20 (4.1) | 21 (12.0) | <0.001 | 17 (4.7) | 40 (14.7) | <0.001 | <0.001 |  |
| CKD | 54 (9.9) | 42 (21.8) | <0.001 | 104 (25.7) | 117 (38.0) | 0.001 | <0.001 |  |
| Stroke | 14 (2.6) | 10 (5.2) | 0.13 | 24 (5.9) | 20 (6.5) | 0.877 | 0.027 |  |
| **Laboratory tests** | | | | | | | |  |
| eGFR, mL/min/1.73 m^2^ | 88.4 (21.8) | 80.0 (32.9) | <0.001 | 74.1 (26.7) | 68.3 (22.9) | 0.003 | <0.001 |  |
| HDLC, mmol/L | 0.96 (0.22) | 0.89 (0.21) | <0.001 | 1.02 (0.24) | 0.93 (0.28) | <0.001 | <0.001 |  |
| LDLC, mmol/L | 2.85 (1.15) | 2.89 (0.98) | 0.646 | 2.57 (0.99) | 2.56 (0.98) | 0.894 | <0.001 |  |
| albumin, g/L | 39.1 (3.2) | 32.2 (2.4) | <0.001 | 38.3 (2.6) | 32.0 (2.5) | <0.001 | <0.001 |  |
| hs-CRP, mg/L | 7.1 (17.0) | 24.6 (40.1) | <0.001 | 4.5 (8.6) | 20.7 (32.8) | <0.001 | <0.001 |  |
| **Medications** | | | | | | | |  |
| ACEI/ARB | 255 (47.0) | 102 (52.8) | 0.186 | 180 (44.4) | 154 (50.0) | 0.162 | 0.208 |  |
| Beta-blockers | 286 (52.7) | 106 (54.9) | 0.649 | 186 (45.9) | 177 (57.5) | 0.003 | 0.015 |  |
| Clopidogrel | 258 (47.5) | 101 (52.3) | 0.286 | 183 (45.2) | 144 (46.8) | 0.734 | 0.435 |  |
| statin | 224 (41.3) | 87 (45.1) | 0.401 | 172 (42.5) | 132 (42.9) | 0.978 | 0.829 |  |
| aspirin | 205 (37.8) | 85 (44.0) | 0.147 | 137 (33.8) | 122 (39.6) | 0.131 | 0.096 |  |

DM= diabetes mellitus; PCI = percutaneous coronary intervention; AMI = acute myocardial infarction; ACS = acute coronary syndrome; EFrEF = ejection fraction reduced heart failure; CKD= Chronic kidney disease; eGFR = estimated glomerular filtration rate; LDL-C =low-density lipoprotein cholesterol; HDL-C= Hight-density lipoprotein cholesterol; hs-CRP = High-Sensitivity C-Reactive Protein; ACEI/ARB = angiotensin-converting enzyme





***Supplementary Figure 1*** *The flow of participants through REICIN cohort*


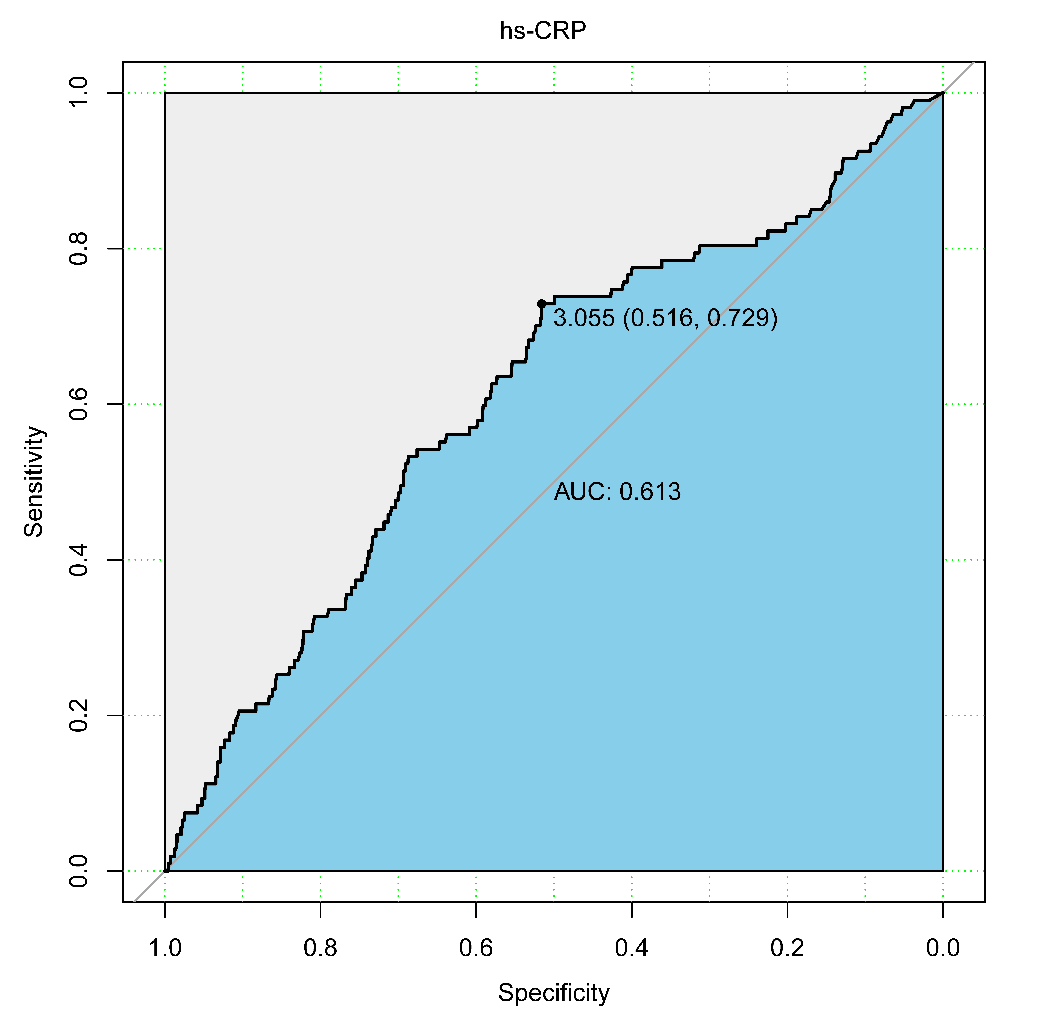
**
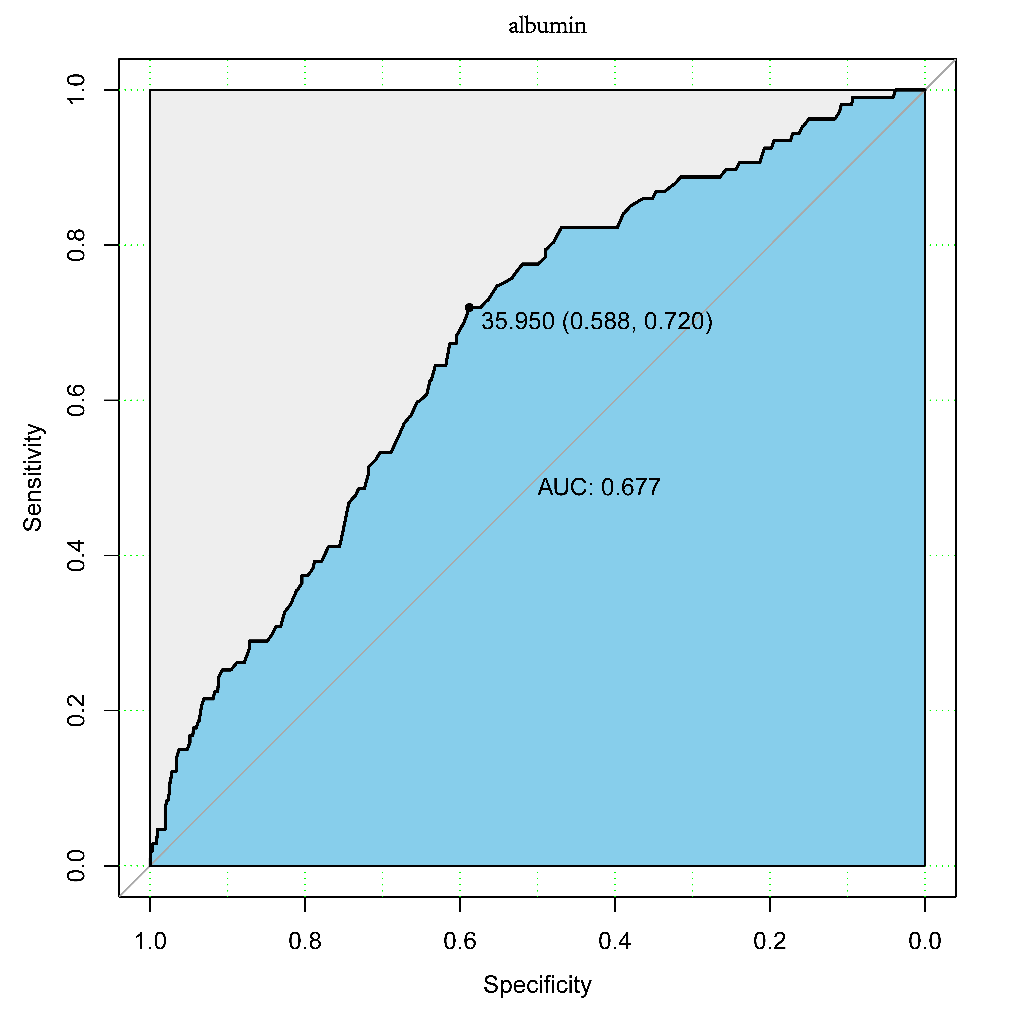
**

***Supplement Figure 2*** *receiver-operator characteristic (ROC) curves*


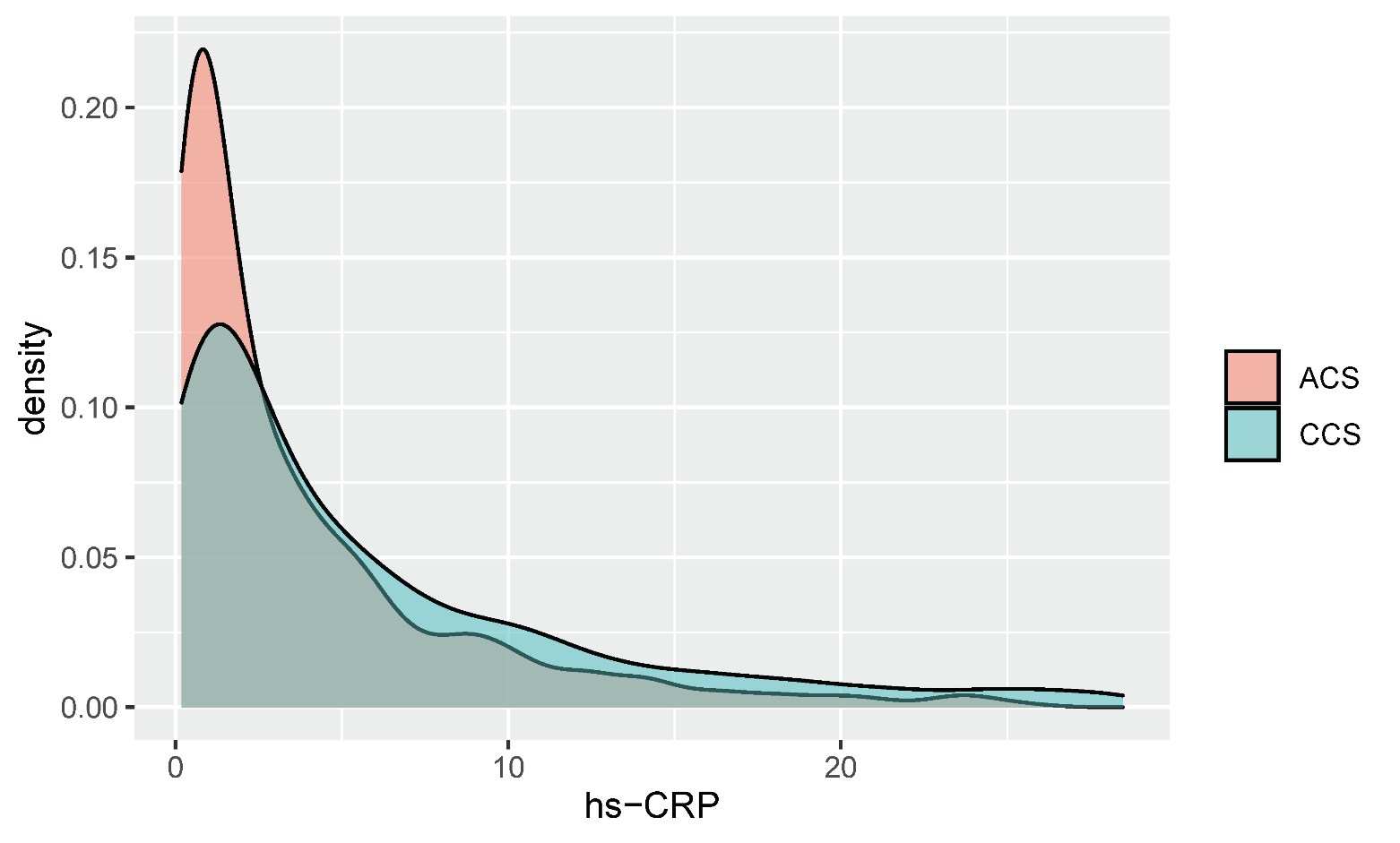


***Supplementary Figure 3*** *Density curve of hs-CRP among ACS and chronic CAD.*





***Supplementary Figure 4*** *Dose-response relationship between hs-CRP tertiles and unadjusted hazard ratios and 95% confidence intervals of long-term all-cause mortality, stratified by status of albumin. ^1^ hs-CRP tertiles (min-max, mg/L): T1(0.18-1.4); T2(1.4-6.0); T3(6.0-194)*P<0.01.*
